# Linking neuronal brain activity to the glucose metabolism

**DOI:** 10.1186/1742-4682-10-50

**Published:** 2013-08-29

**Authors:** Britta Göbel, Kerstin M Oltmanns, Matthias Chung

**Affiliations:** 1 Institute of Mathematics and Image Computing, University of Lübeck, Ratzeburger Allee 160, 23538 Lübeck, Germany; 2 Department of Psychiatry and Psychotherapy, Division of Psychoneurobiology, University of Lübeck, Ratzeburger Allee 160, 23538 Lübeck, Germany; 3 Department of Mathematics, Virginia Tech, 225 Stanger Street, 474 McBryde Hall, Blacksburg, VA 24061, USA

**Keywords:** Energy metabolism, Physiological modeling, Dynamical system, Energy homeostasis, Neuronal brain activity

## Abstract

**Background:**

Energy homeostasis ensures the functionality of the entire organism. The human brain as a missing link in the global regulation of the complex whole body energy metabolism is subject to recent investigation. The goal of this study is to gain insight into the influence of neuronal brain activity on cerebral and peripheral energy metabolism. In particular, the tight link between brain energy supply and metabolic responses of the organism is of interest. We aim to identifying regulatory elements of the human brain in the whole body energy homeostasis.

**Methods:**

First, we introduce a general mathematical model describing the human whole body energy metabolism. It takes into account the two central roles of the brain in terms of energy metabolism. The brain is considered as energy consumer as well as regulatory instance. Secondly, we validate our mathematical model by experimental data. Cerebral high-energy phosphate content and peripheral glucose metabolism are measured in healthy men upon neuronal activation induced by transcranial direct current stimulation versus sham stimulation. By parameter estimation we identify model parameters that provide insight into underlying neurophysiological processes. Identified parameters reveal effects of neuronal activity on regulatory mechanisms of systemic glucose metabolism.

**Results:**

Our examinations support the view that the brain increases its glucose supply upon neuronal activation. The results indicate that the brain supplies itself with energy according to its needs, and preeminence of cerebral energy supply is reflected. This mechanism ensures balanced cerebral energy homeostasis.

**Conclusions:**

The hypothesis of the central role of the brain in whole body energy homeostasis as active controller is supported.

## Background

Regulation of the energy metabolism is crucial to ensure functionality of the human organism. However, the interactions of numerous energy metabolites and neuroendocrine mechanisms in the complex regulation are still not completely understood.

So far, there exist various theoretical approaches to explain the regulation of the human energy metabolism. Two traditional concepts are called the glucostatic theory [1] and the lipostatic theory [2], in which blood glucose or lipids, respectively, are the regulated quantities. The decisive role of the brain in the global regulation of the complex whole body energy homeostasis is subject to current research activities. Studies suggest a priority of cerebral energy supply, while all organs in the organism compete for available energy resources [3-7].

The “Selfish Brain Theory” provides a new approach to explain the regulation of the human whole body energy metabolism [8,9]. This theory includes regulatory mechanisms of both the glucostatic and the lipostatic theory and extends them by the administrative position of the brain. The brain is not an isolated organ within the organism but, right to the contrary, the superordinate administrative instance within the hierarchy of all organismic processes. Concomitantly, the brain is a heavy energy consumer with an uptake of up to 20% of total glucose on daily average [10]. According to the Selfish Brain Theory, the brain has two principal mechanisms to provide itself with sufficient energy, on the one hand, the regulation of external food intake and on the other hand, the allocation of intrinsic energy resources from the body periphery. Under conditions of low cerebral energy levels, glucose transport across the blood brain barrier seems elevated as indicated by increased levels of the cerebral high-energy phosphate content [5]. Accordingly, the energy transport into peripheral stores is suppressed. With low blood insulin concentrations, glucose is allocated to the brain since glucose transport across the blood brain barrier, in contrast to peripheral organs and tissues, is mainly insulin-independent.

Hence, identifying control mechanisms of the brain energy homeostasis is a major goal in obtaining a systemic understanding of the human overall energy metabolism and thereby providing insight into pathological regulation [8]. This motivates the investigation of the tight link between neuronal brain activity and systemic metabolic responses of the organism.

In the present study, we aim to gain specific information about the regulatory elements of the human brain in the systemic energy homeostasis. Therefore, we combine mathematical modeling and experimental data. In our novel approach, the integrative behavior of the human whole body energy metabolism is mathematically modeled in a compact dynamical system [11,12]. This model takes into account the central roles of the brain with respect to the systemic energy homeostasis. That is, the brain is considered as regulatory instance and as energy consumer. Energy fluxes and their control signals, such as glucose fluxes and hormonal signals, are integrated in the dynamical system. The peripheral hormone insulin is regarded not only as local signal but also as key feedback signal to the brain [13-15]. Hence, in the mathematical model we integrate the competition for energy between brain and body periphery. There exist numerous mathematical models of human glucose metabolism, e.g., [16-22]. However, in our novel approach we formulate the cerebral energy content in terms of high-energy phosphate levels comprising energy metabolites that emerge from several energy supplying substrates such as glucose and lactate. The novelty of our approach lies in combining a brain centered mathematical model with experimental data of an euglycemic-hyperinsulinemic clamp to reveal systemic information of the brain energy metabolism. Our approach includes a new parameter estimation method to unfold major features of the energy regulation.

A close relation between brain energy homeostasis and systemic glucose metabolism has been suggested several times, e.g., [7,23,24]. In an experimental study [25], the close link between neuronal brain activity and subsequent metabolic responses of the human organism was for the first time investigated in a human in vivo approach. In order to clarify underlying mechanisms in the context of this experimental study, we solve the inverse problem and identify model parameters of the dynamical system. Thereby, we aim to gain specific information about the relationship between neuronal brain activity, cerebral energy homeostasis, and peripheral metabolism.

In the following Material and methods section, we describe the experimental study in Experimental study section and introduce the mathematical model in Brain-centered energy metabolism model section. Parameter estimation methods are presented in Parameter identification section and Parameter identification setup section. Results of the parameter identification are investigated in Results section. We close with a discussion and a brief outlook in Conclusions section.

## Material and methods

### Experimental study

**Figure 1:**
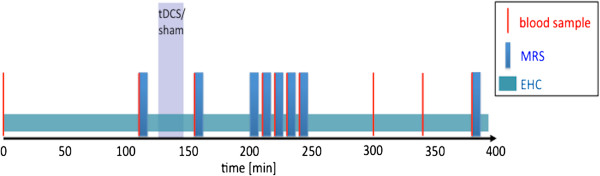
**Design of the experimental study.** Blood samples and ^31^phosphorus magnetic resonance spectra (MRS) were regularly taken under steady state conditions of an euglycemic hyperinsulinemic clamp (EHC) to monitor cerebral and peripheral energy metabolism. Transcranial direct current stimulation (tDCS) or sham stimulation, respectively, were induced to affect neuronal brain activity.

The goal of our experimental study is to investigate the close link between neuronal brain activity and subsequent metabolic responses of the human organism at a systemic level. In [25], the methods and results of the experimental study are described in detail. The study design is depicted in Figure 1.

In a randomized sham-controlled crossover design, a homogeneous group of 15 healthy young male volunteers with a body mass index of 23.2 ± 0.38 kg/m^2^ is examined. Neuronal brain activity is stimulated by transcranial direct current stimulation (tDCS) during the time interval *t* ∈ [125, 145] (minutes). Transcranial stimulation of the brain causes transient effects on motor cortical excitability outlasting the stimulation period [26]. Sham stimulation serves as control condition. For sham stimulation, electrodes are placed at the same site without current stimulation. TDCS-induced effects on cerebral energy metabolism and systemic glucose regulation are measured. The study is carried out in accordance with the Declaration of Helsinki (2000) of the World Medical Association and has been approved by the ethics committee of the University of Lübeck. Each participant gave written informed consent.

^31^Phosphorus magnetic resonance spectroscopy (^31^P-MRS) allows performing non-invasive in vivo measurements of brain metabolites that are centrally involved in the energy metabolism. Phosphate metabolites such as adenosinetriphosphate (ATP), i.e., the sum of α-, β-, and γ-ATP, as well as phosphocreatine are measured in the cortex reflecting the overall high-energy phosphate turnover [27]. Here, the ratio of ATP and inorganic phosphate (Pi) is evaluated as an indicator of the intracellular energy status [25,28]. ^31^P-MR spectra are measured at times *t* = 115, 160, 205, 215, 225, 235, 245, 385 (minutes).

During euglycemic-hyperinsulinemic clamping, an insulin infusion at the predetermined fixed dosage of 1.5 mU (kg min)^−1^ and a variable glucose infusion are administered in order to reach stable plasma glucose concentrations between 4.5 and 5.5 mmol/l. Under steady-state conditions of euglycemia, the glucose infusion rate equals glucose uptake by all tissues in the body [29] and is therefore a measure of glucose tolerance. To monitor the peripheral glucose metabolism, blood samples of glucose and insulin are regularly taken at times *t* = 0, 110, 155, 210, 220, 230, 240, 300, 340, 380 (minutes).

**Figure 2:**
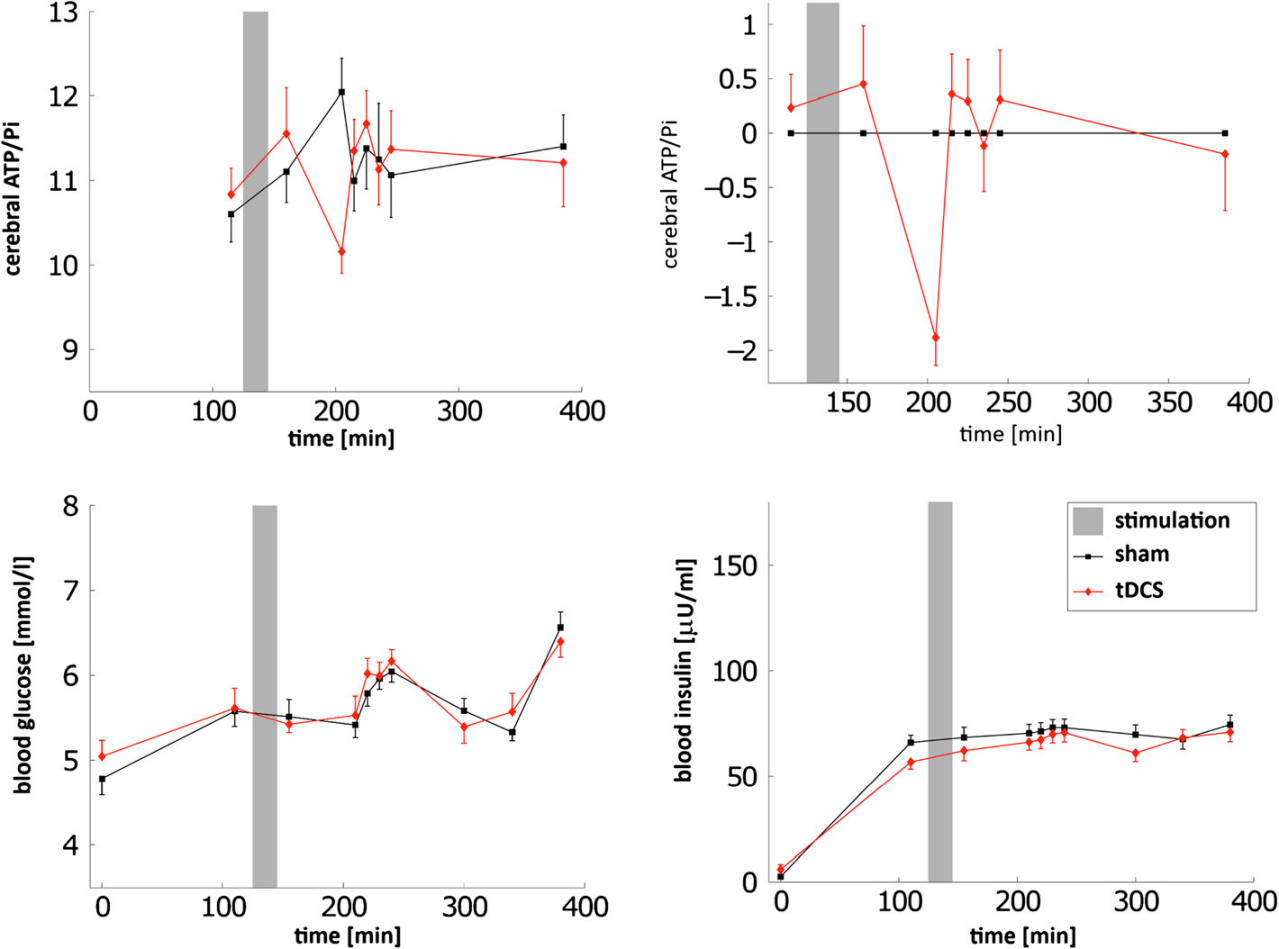
**Experimental data.** Effects of transcranial direct current stimulation (red) on cerebral ATP/Pi, blood glucose, and insulin during a hyperinsulinemic-euglycemic glucose clamp condition as compared with the sham stimulation (black). The top right figure shows relative changes of cerebral ATP/Pi in proportion to matched sham condition values. Data show a biphasic course with an initial drop by trend followed by a rise. The gray background indicates the stimulation interval. Data represent mean values +/- standard error of mean (Figure reproduced from [25]).

While blood glucose and insulin concentrations do not differ between conditions, overall cerebral high-energy phosphate measurements display a biphasic course upon tDCS as compared with the sham condition, see [25] and Figure 2. An initial energetic drop in the ATP/Pi ratio upon tDCS is observed. Subsequent ^31^P-MR spectra reveal a rapid increase above the control condition followed by a return of the ATP/Pi ratio to baseline levels. Glucose infusion rates show the same biphasic response to tDCS indicating that transcranial stimulation improves systemic glucose tolerance [29-31]. Measurements of the hypothalamus-pituitary-adrenal (HPA) hormonal system reveal decreasing concentrations of circulating stress hormones such as cortisol upon tDCS (compare [25]).

The experimental data demonstrate that transcranial brain stimulation not only evokes alterations in local neuronal processes but also clearly influences brain energy homeostasis and peripheral metabolic systems regulated by the brain [25]. Hence, manipulating brain activity by tDCS affects peripheral metabolic regulation such as the glucose metabolism and related neuroendocrine mediators. Effects of tDCS on cerebral ATP/Pi, blood glucose, and insulin as compared with the sham stimulation are shown in Figure 2.

Nevertheless, the mechanisms underlying these experimental observations remain unknown. Concerning the specific mechanisms by which neuronal excitation, via a drop in high-energy phosphate content, improves glucose tolerance, one can only speculate at this point.

The objective of the following mathematical analysis is to gain insight into physiological mechanisms underlying the effects of brain stimulation on cerebral and peripheral energy metabolism. In order to clarify the underlying mechanisms in this context, we combine experimental data with the mathematical model introduced in the following subsection.

### Brain-centered energy metabolism model

Physiological processes may be described by systems of ordinary differential equations,

(1) $$\frac{\mathrm{dy}}{\mathrm{dt}}\left(t,p\right)=f\left(t,y,p\right)\;\mathrm{with}\;y\left(0\right)=y_{0},$$

where $\;y:R\times R^{m}\to R^{n}$ and $f:R\times R^{n}\times R^{m}\to R^{n}$ are time-depending functions with *t*∈[0,*T*]. Here, *y*_0_ denotes the initial conditions. We collect unknown model parameters to be estimated in the vector *p* = (*p*_1_, …, *p*_*m*_)^⊤^. In the following, given parameters regarded as constant are assembled in a vector *c*.

A collection of mathematical models of this kind describing interactions of main components of the human glucose metabolism can found in the book of Chee and Fernando [17]. Most of these models are based on the glucostatic or lipostatic theory. Exemplarily, one could mention the well-known Minimal Model [32], the Ackerman Model [14], or more recently models published in [19,21]. Additionally, mathematical models developed in the context of the Selfish Brain Theory can be found in the review paper by Chung and Göbel [33].

**Figure 3:**
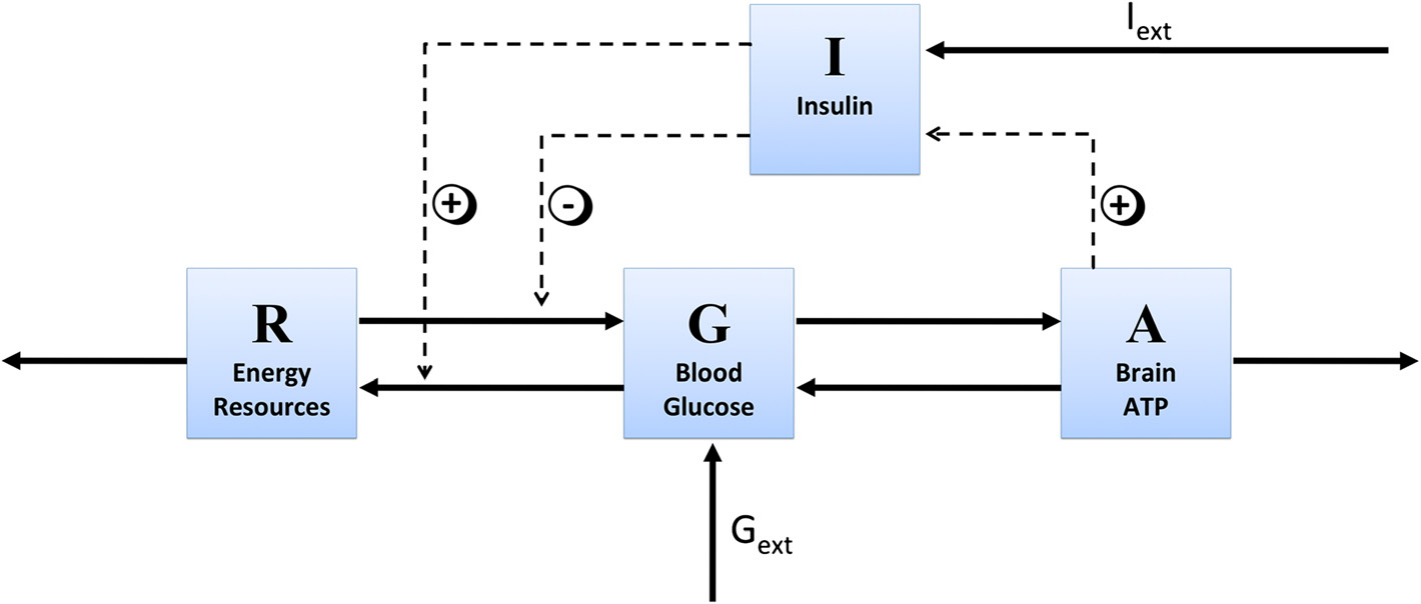
**Illustration of the brain centered energy model (2).** Energy fluxes between compartments (solid) and control signals directing energy fluxes within the organism (dashed).

Here, we regard a mathematical model of the human whole body energy metabolism considering the brain not only as energy consumer but more importantly as a superordinate controller, compare [12]. The model includes energy metabolites in separated compartments, energy fluxes between these compartments, and signals directing energy fluxes within the organism, see Figure 3.

The brain-centered model of the energy metabolism is given by the system of four ordinary differential equations,

(2) $$\begin{array}{l} \frac{\mathrm{dA}}{\mathrm{dt}}=p_{1}\frac{G}{A}-p_{4,}\\ \frac{\mathrm{dG}}{\mathrm{dt}}=-p_{1}\frac{G}{A}-c_{1}\mathrm{GI}+p_{2}\frac{R}{G}+G_{\mathrm{ext}},\\ \frac{\mathrm{dI}}{\mathrm{dt}}=p_{3}A-c_{2}I+I_{\mathrm{ext}},\\ \frac{\mathrm{dR}}{\mathrm{dt}}=c_{1}\mathrm{GI}-p_{2}\frac{R}{G}-p_{5}. \end{array}$$

Cerebral energy content, i.e., high-energy phosphates in the brain, is denoted by *A*. Furthermore, *G* is the blood glucose concentration, and *R* specifies energy resources in the body, which comprise available energy reserves foremost in liver, muscle, and fat tissue. Note, various types of energy, such as fat, glycogen, and glucose, are combined in the energy resources compartment. In addition to energy metabolites, the model contains the control signal, identified as blood insulin concentration.

Conceptually, our model bases on conservation of energy. In general, stimulatory influences are modeled as proportional relations and inversely proportional relations describe prohibitive influences. The glucose flux from the blood into the brain crossing the blood brain barrier is proportional to intensified by a factor *p*_1_ [M s^−1^]. This factor quantifies the glucose flux rate from the blood into the brain across the blood brain barrier. Suppose the cerebral energy content *A* is low, the facilitated diffusion process from the blood compartment into the brain via the passive glucose transporter GLUT1 is accelerated. Contrary, high levels of *A* inhibit this flux. This can be seen as “energy on demand” of the brain. This concept has been published in [12]. Glucose *G* needs to be available in the blood compartment to reach the brain.

Our model combines energy resources and metabolites, such as glycogen, glucose and lactate, in the compartment *G*. The energy flux from the resources into the blood compartment, which is composed of several sub-mechanisms, is proportional to the energy resources *R* and anti-proportional to the actual blood glucose concentration *G* with a flux rate with a factor *p*_2_ [M s^−1^], i.e., *p*_2_*R / G*. This flux includes endogenous glucose production by the liver amongst others.

The hormone insulin acts not only as local response to the blood glucose concentration. Moreover, it is regarded as central feedback signal of the brain with an insulin secretion factor *p*_3_ [s^−1^]. Notice that with low cerebral energy, ventromedial hypothalamic centers inhibit pancreatic insulin secretion [34-36]. Being an anorexigenic hormone, peripherally secreted insulin is a key feedback signal to the brain reducing food intake and systemic glucose uptake [13,15]. Energy consumption of the brain and energy consumption by the periphery are denoted by *p*_4_ [M s^−1^] and by *p*_5_ [M s^−1^].

Insulin-dependent glucose uptake from the blood into the energy resources compartment is modeled by *c*_1_*GI* with a parameter *c*_1_ [(M s)^−1^]. This flux mainly comprises glucose uptake into the peripheral stores, i.e., muscle and fat tissue. To accelerate this flux, glucose and insulin need to be available in the blood at the same time in order to activate glucose uptake via the insulin-dependent glucose transporter GLUT4.

Degradation of insulin is supposed to be of first order with the insulin degradation rate *c*_2_ [s^−1^]. External glucose infusion is denoted by *G*_*ext*_(*t*) [M s^−1^], insulin infusion is *I*_*ext*_(*t*) [M s^−1^].

To meet the simplified notation from Equation (1) we collect the state variables *y* = (*A*, *G*, *I*, *R*)^⊤^ and the parameters *p* = (*p*_1_, …, *p*_5_)^⊤^. The constants *c* = (*c*_1_, *c*_2_)^⊤^ and time-dependent external infusions *G*_*ext*_, *I*_*ext*_ are not explicitly shown in (1). Notice that all parameters, constants, and states are non-negative. Model properties he been investigated in detail and it has been shown that the model realistically reproduces qualitative and quantitative behavior of the whole body energy metabolism even for a large class of physiological interventions (see [11,12] for details).

To accommodate the characteristics of the experimental study, the dynamical system (2) slightly differs from the model introduced in [12]. First, glucose and insulin infusions are administered. Secondly, ingestion of food is neglected since no food intake occurs during the examinations.

### Parameter identification

In the following, we introduce the general technique to estimate the model parameters *p*. The solution *y* of the dynamical system (2) varies with respect to the model parameters *p*. In order to validate and implement model predictions, the mathematical model needs to be compared to experimental data. Here, we analyze the model behavior in identifying the unknown model parameters *p* = (*p*_1_, …, *p*_5_)^⊤^ of our dynamical system in Brain-centered energy metabolism model section using experimental data presented in Experimental study section.

In general, parameter identification problems for ordinary differential equations can be stated as follows

(3) $$\begin{array}{l} \min_{\left(p,y_{0}\right)}\frac{1}{2}∥\sigma^{-2}\left(y\left(\tau ,p\right)-d\right){∥}_{2}^{2}+S\left(\lambda ,p\right)\;\\ \;\mathrm{subject}\;\mathrm{to}\;\frac{\mathrm{dy}}{\mathrm{dt}}=f\left(t,y,p\right)\;\mathrm{and}\;y\left(\tau_{1}\right)=y_{0}. \end{array}$$

Equation (3) states a classical constrained optimization problem, where constraints are given by an initial value problem [37]. With ∥·∥_2_ denoting the Euclidian norm, we minimize the distance between the model solution at the times *τ* = (*τ*_1_, …, *τ*_*k*_)^⊤^, where the data are measured and given data $d={\left(d_{1},\ldots ,d_{k}\right)}^{⊤}\in R^{k}$; the minimization is constrained by the validity of the mathematical model. With $\sigma^{2}\in R$ denote the variance of the data assumed to be independent and identically distributed. The term *S*(λ,*p*) is a convex regularization inducing prior knowledge on the parameters for which we will provide details later. For convenience, we assume the model function *f* to be continuously differentiable. Note that the optimization problem (3) corresponds to the maximum likelihood estimator including a prior and is a standard formulation of a parameter identification problem [38]. Note that estimator (3) infers that the errors are independently and identically normal distributed.

We seek to find $\hat{p}\in R^{m}$ minimizing Equation (3). The optimization problem (3) can only be targeted by numerical optimization methods. Problem (3) is a typical inverse problem since data and model are given and we aim to identify the model parameters. This inverse problem is well known to experimentalists and various methods have been established to solve such type of parameter estimation problems. Most commonly used are single and multiple shooting methods. Both methods face certain advantages and disadvantages. Single shooting methods are easy to implement but are not robust to initial guesses of the model parameters and optimization algorithms are likely to fail or to find “non-optimal” local minimizer. Multiple shooting methods are by far more robust and are shown to face better convergence. However, multiple shooting methods solve constrained optimization problems, which dramatically increase the algorithmic complexity [37,39].

Here, we follow an approach similar to methods proposed by Ramsay et al. [40], Chung and Haber [41], or Poyton et al. [39]. Equation (3) can be restated as

(4) $$\begin{array}{l} \min_{\left(p,y_{0}\right)}\frac{1}{2}∥\sigma^{-2}\left(y\left(\tau ,p\right)-d\right){∥}_{2}^{2}+S\left(\lambda ,p\right)\\ \mathrm{subject}\;\mathrm{to}\;{∥\frac{\mathrm{dy}}{\mathrm{dt}}-f\left(t,y,p\right)∥}^{2}=0\;\mathrm{and}\;y\left(\tau_{1}\right)=y_{0}. \end{array}$$

The equivalence of optimization problem (3) and (4) stays true for any appropriate integral norm ∙ (here we choose the *L*^2^ –norm). Constrained optimization problems such as (4) are commonly approximated by performing a Lagrangian relaxation [38]. We get:

(5) $$\begin{array}{l} \min_{\left(p,y_{0}\right)}\frac{1}{2}∥\sigma^{-2}\left(y\left(\tau ,p\right)-d\right){∥}_{2}^{2}+\frac{\alpha }{2}\;{∥\frac{\mathrm{dy}}{\mathrm{dt}}-f\left(t,y,p\right)∥}^{2}+S\left(\lambda ,p\right)\\ \mathrm{subject}\;\mathrm{to}\;y\left(\tau_{1}\right)=y_{0}, \end{array}$$

with the Lagrangian multiplier *a* ≥ 0. Notice that for increasing *a* unconstrained optimization problem (5) becomes optimization problem (4). Next, we choose a standard discretize-then-optimize approach to solve problem (5), numerically. We let $u={\left(u_{1},\ldots ,u_{k}\right)}^{⊤}\in R^{k}$ be a approximation of *y* at the data points *τ*_1_, …, *τ*_*k*_ and *D*_*t*_ be a finite differences operator approximating *dy* / *dt* at *τ*_1_, …, *τ*_*k*_, then we can restate the optimization problem (5) as the discretized and unconstrained optimization problem.

(6) $$\min_{\left(p,u\right)}\Phi \left(p,u\right)=\frac{1}{2}∥\sigma^{-2}\left(u-d\right){∥}_{2}^{2}+\frac{\alpha }{2}∥D_{t}\;u-f\left(\tau ,u,p\right){∥}_{2}^{2}+S\left(\lambda ,p\right).$$

Notice that we can neglect the remaining constraint in (5) since *y*_0_ = *u*_1_ is already included in the search parameters and is therefore always fulfilled. Equation (6) describes the general parameter estimation framework. This optimization problem has the advantages that it is robust and the unconstrained nature allows to use fast gradient-based methods, for details see [40,41]. Notice that if the data points *τ*_1_, …, *τ*_*k*_ are t dense, one may want to utilize a spline function *s* with knots τ and coefficients *q* at dense points $\xi ={\left(\xi_{1},\ldots ,\xi_{l}\right)}^{⊤}$ to capture the dynamic of the differential equation. Then Equation (6) reads

(7) $$\min_{\left(p,q\right)}\Phi \left(p,q\right)=\frac{1}{2}∥\sigma^{-2}\left(s\left(\tau ,q\right)-d\right){∥}_{2}^{2}+\frac{\alpha }{2}∥s'\left(\xi ,q\right)-f\left(\xi ,s\left(\xi ,q\right),p\right){∥}_{2}^{2}+S\left(\lambda ,p\right).$$

One further choice to make is choosing the regularization term *S*(λ,*p*). A most common choice for the regularization term is

$$S\left(\lambda ,p\right)=\frac{\lambda }{2}\sum {\left(\frac{p_{i}-{\bar{p}}_{i}}{{\bar{p}}_{i}}\right)}^{2},$$

where $\bar{p}\in R^{m}$ is a given parameter representing physiological parameter values and λ ≥ 0. With this we have established a parameter estimation method to tackle the model of Brain-centered energy metabolism model section.

### Parameter identification setup

Next, we present the parameter estimation setup for the model in Section 2.2 with the given data from Section 2.1. As derived in Section 2.2, all parameters of our model (2) have a physiological interpretation. Since insulin is at hyperphysiological levels in our experimental examination we will consider the dependent parameters *c*_1_ and *c*_2_ to be constant. Flakoll PJ, Wentzel LS, Rice DE, Hill JO and Abumrad NN [42] quantify the insulin-dependent whole body glucose uptake corresponding to *c*_1_ ≈ 0.06 (pM min)^−1^. Information about insulin clearance is given by [43] resulting in *c*_2_ ≈ 1.4 min^−1^. This yields the fixed parameter values *c* = (*c*_1,_*c*_2_) = (0.06, 1.4)^⊤^.

For the parameter $\bar{p}={\left({\bar{p}}_{1},\ldots ,{\bar{p}}_{5}\right)}^{⊤},$ we choose physiologically relevant parameter values gathered from the literature. We use ${\bar{p}}_{1}\approx 0.15$ mM/min for glucose transport rate across the blood brain barrier [44]. Baron AD and Clark MG [45] specify the glucose flux between peripheral stores and blood with ${\bar{p}}_{2}\approx 0.6\;$ mM/min. For the insulin secretion rate, we choose ${\bar{p}}_{3}\approx 20$ min^−1^, see [46]. The work by Flakoll PJ, Wentzel LS, Rice DE, Hill JO and Abumrad NN [42] provides the maximal rate of glucose utilization. This grants an insight into the peripheral energy consumption ${\bar{p}}_{5}\approx 0.7$ mM/min and cerebral energy consumption ${\bar{p}}_{4}\approx 0.175$ mM/min since the brain uses up to 20% of total body glucose [10]. Hence, we choose the regularization value $\bar{p}={\left(0.15,0.6,20,0.175,0.7\right)}^{⊤}$.

In order to establish global convergence, we choose a Monte Carlo sampling technique of the initial guess *p*_0_. We pick 500 randomly chosen normally distributed samples with mean $\bar{p}$. For each sample we calculate the minimizer and choose the overall minimizer $\bar{p}$ to be the minimizer of the objective function *Φ*, see Equation (4).

To solve the optimization problem (4) numerically we use a Gauss-Newton method with Armijo line search, see [38]. The regularization parameters *a* and *λ* are set to *a* = 5 10^−3^ and *λ* = 10^−7^ for our numerical investigations. By empirical observations these values lead to a good balance between under- and overfitting.

## Results

### Model validation

The goal of this section is to show that parameter values of our dynamical system (2) can be chosen such that the resulting mathematical model is able to approximate the experimental measurements of the study described in Section 2.1. To validate the mathematical model, we first identify the model parameters by mean values of the experimental data for cerebral ATP/Pi, blood glucose, and insulin concentration in the sham stimulation condition. Note, for the energy resources compartment no experimental data are available, and we arbitrarily set *R* = 50 [mmol/l]. We let the corresponding variance σ^2^ = 1000 be fairly high, which allows the optimization method to pick adequately different values for *R* without contributing much to the function *Φ*. Thereby, we obtain the minimizer $\hat{p}={\left(0.0049,0.0683,11.3768,0.0025,0.8331\right)}^{⊤}$ with objective function value *Φ =* 6.7611 10^−5^ for the placebo group.

**Figure 4:**
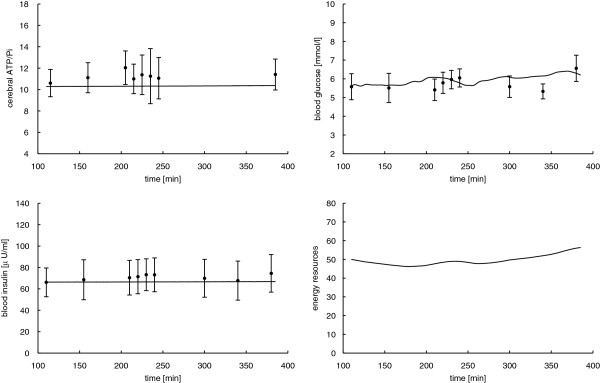
**Experimental data (error bars) versus mathematical model (solid lines) for the mean sham stimulation data.** Error bars show mean values and respective standard errors of the mean of cerebral ATP/Pi, blood glucose, and insulin levels. For energy resources, no experimental data are available. Solid lines represent model predictions with the estimated parameter values $\hat{p}={\left(0.0040,0.0683,11.3768,0.0025,0.8331\right)}^{⊤}\;$ with objective function Φ = 6.7611 · 10^−5^ and relative mean squared error $\theta \left(\hat{p}\right)=$ 7.6012 · 10^−3^.

In order to test the accuracy of the estimation, we calculate the distance between data and calculated model by the relative mean squared error

(8) $$\theta \left(p\right)=\frac{1}{K}\overset{K}{\underset{k=1}{\sum }}{\left(\frac{y_{k}-d_{k}}{d_{k}}\right)}^{2},$$

where *K* is the number of data points. The vector *d* contains the experimental data of cerebral high-energy phosphates, blood glucose, and insulin concentrations, (experimental data and model prediction are shown in Figure 4). The vector *y* specifies the respective solution of the dynamical system (2) evaluated at the data time points in the interval [110, 385] min. We obtain the relative mean squared error $\theta \left(\hat{p}\right)=$ 7.6012 · 10^−3^.

**Figure 5:**
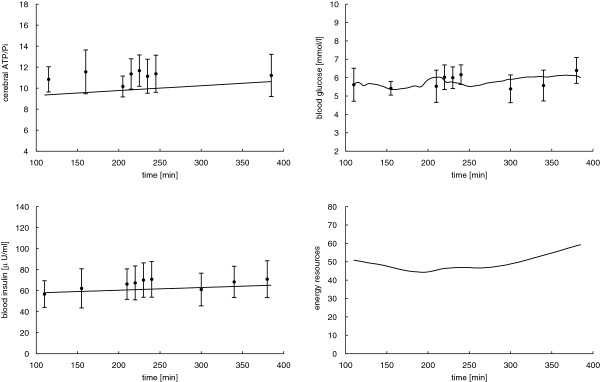
**Experimental data (error bars) versus mathematical model (solid lines) for the mean tDCS stimulation data.** Error bars show mean values and respective standard errors of the mean of cerebral ATP/Pi, blood glucose, and insulin levels. For energy resources, no experimental data are available. Solid lines represent model predictions with the estimated parameter values $\hat{p}={\left(0.0092,0.0474,10.8068,0.0006,0.8507\right)}^{⊤}$ with objective function value with Φ = 2.0410 · 10^−4^ and relative mean squared error $\theta \left(\hat{p}\right)=$ 9.6891 · 10^−3^.

The parameter estimates for the mean data from the tDCS intervention read $\hat{p}={\left(0.0092,0.0474,10.8068,0.0006,0.8507\right)}^{⊤}$, with *Φ* = 2.0410 10^−4^ and $\theta \left(\hat{p}\right)=$ 9.6891 · 10^−3^ and are illustrated in Figure 5.

Since we conduct parameter identification with 500 random initial values *p*_0_ as well as the values *Φ* and *θ* are small, it is likely that the estimated values $\hat{p}$ are the global minimum. Therefore, the model parameters can be chosen such that the mathematical curves closely approximate the data. Note that the estimated parameter values $\hat{p}$ stay not too far from the initial guesses $\bar{p}$, taken from the literature [4,17,39,40]. However, uncertainties in the data may amplify uncertainties in the estimated parameters.

With the estimated parameter values $\hat{p}$ we solve the forward problem. Figures 4 and 5 show the model curves *y* = (*A*, *G*, *I*, *R*)^⊤^ compared with experimental data for sham and tDCS interventions. The model predictions closely approximate the measurements. Ideally a glycemic-hyperinsulinemic clamp generates constant blood glucose and plasma insulin levels. Constant glucose and insulin levels by themselves do not contribute much insight to the parameter estimation. However, the glucose and insulin fluxes *G*_*ext*_ and *I*_*ext*_ are recorded and are included in the parameter estimation. Note that blood glucose predictions are slightly unsteady due to the external glucose infusion.

**Figure 6:**
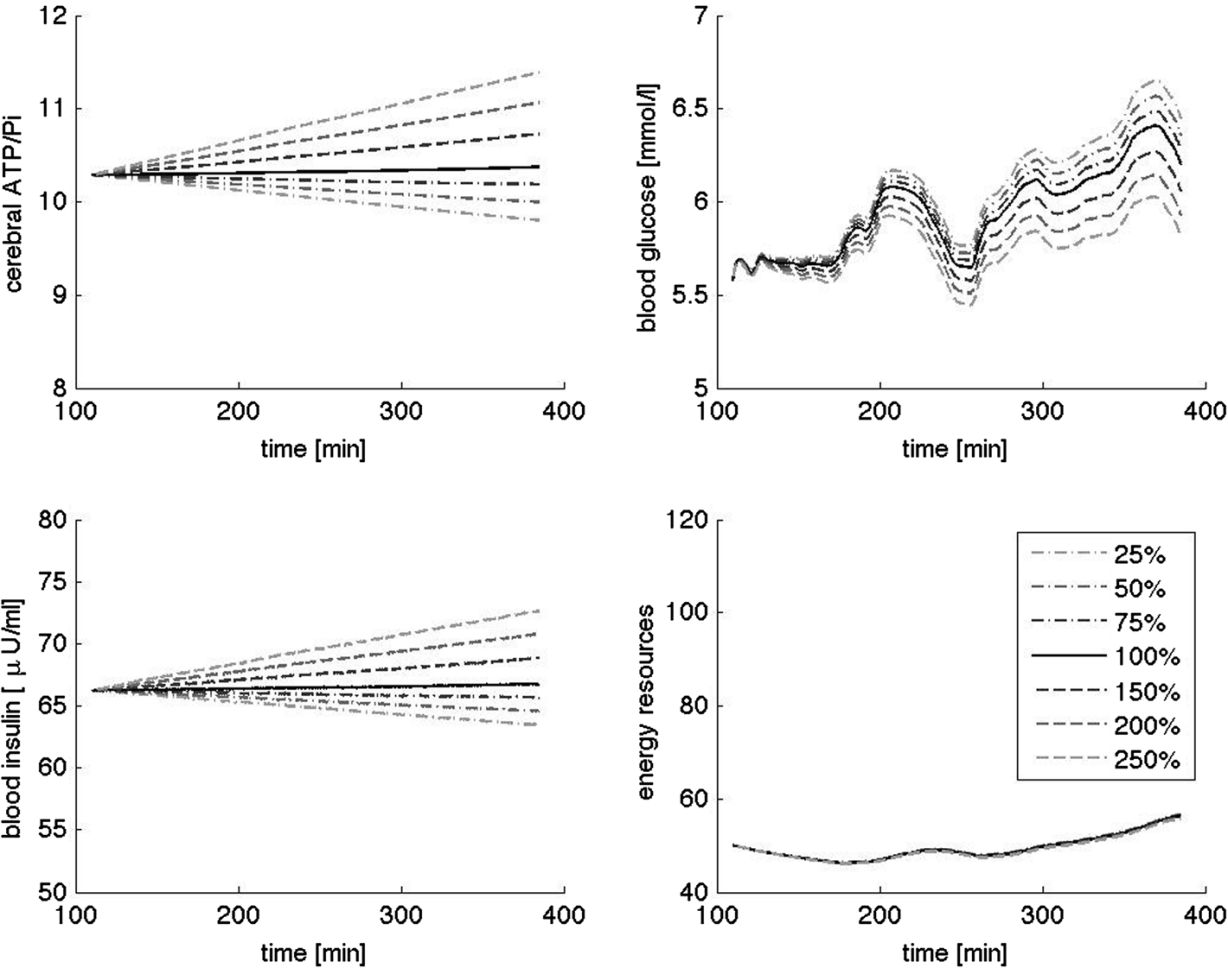
**Effects of parameter modifications on model predictions of cerebral ATP, blood glucose, and insulin concentrations as well as on energy resources.** Stepwise increase in cerebral glucose uptake p_1_ by 150, 200, and 250% leads to marked elevations of predicted brain ATP and blood insulin as well as reduced blood glucose profiles. Hence, the predictions are very sensitive to changes in this parameter.

**Figure 7:**
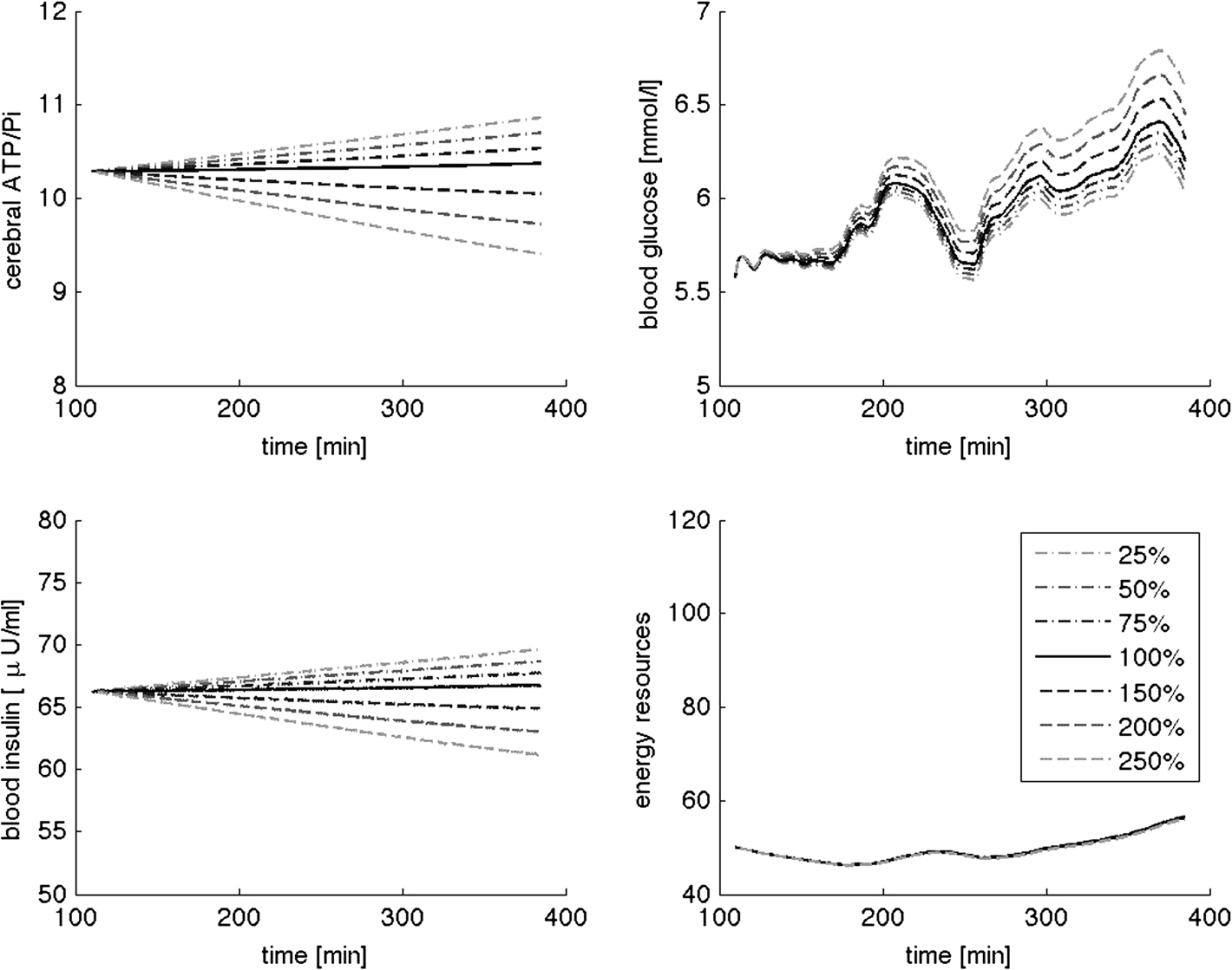
**Modifying cerebral energy consumption p**_**4 **_**affects the predicted profiles of cerebral ATP, blood glucose, and insulin concentrations in a sensitive manner.**

To validate the mathematical model, we perform a sensitivity analysis. The results of the sensitivity analysis for the two central parameters *p*_1_ and *p*_4_ are illustrated in Figures 6 and 7, respectively. We find that model predictions of cerebral ATP *A*, blood glucose *G*, and insulin *I* are sensitive to changes in these parameters. Modifying model parameters *p*_2_, *p*_3,_*p*_5_ and initial values *y*_0_ also affects the predicted profiles, indicating their sensitivity to changes in these parameters as well (analysis not shown). Energy resources *R* are sensitively affected mainly by variations in peripheral energy consumption *p*_5_.

### Effects of tDCS on underlying physiological mechanisms

**Figure 8:**
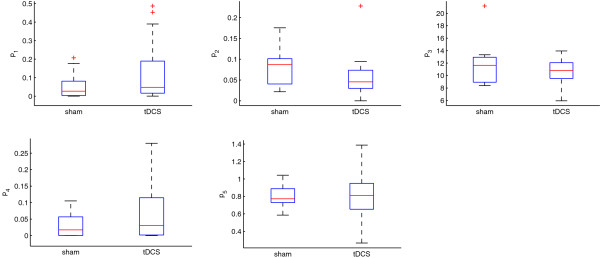
Box-and-whisker diagrams of identified parameter values for current and sham stimulation, respectively.

While Section 3.2 considers the population of placebo and tDCS data, we next investigate each individual data set using the same settings as before. We identify the parameters $\;{\hat{p}}_{1},\ldots ,{\hat{p}}_{5}\;$ for each volunteer participating in the experimental study separately. Thereby, we are able to identify reasonable parameter values for each subject. Box-and-whisker diagrams of the estimated parameter values are shown in Figure 8.

We statistically investigate the effects of tDCS on estimated parameter values. Each model parameter has a physiological interpretation. Thereby, our examinations provide insight into underlying physiological mechanisms that are not measurable in the experimental study.

We identify outliers $\hat{p}\_i$ below quartile *Q*_1_ and above quartile *Q*_3_ by ${\hat{p}}_{i}<Q_{1}-1.5\left(Q_{3}-Q_{1}\right)$ and ${\hat{p}}_{i}>Q_{1}+1.5\left(Q_{3}-Q_{1}\right)$, respectively (see red plus in Figure 8). Individuals with extreme outliers are plotted individually. Mean values and standard deviations of the estimated parameters $\hat{p}$ are listed in Table 1.

**Table 1:** Mean estimated parameter values and respective standard errors of mean

	***sham***	***tDCS***
${\hat{p}}_{1}$	0.0555 ± 0.0709	0.1362 ± 0.1752
${\hat{p}}_{2}$	0.0785 ± 0.0463	0.0583 ± 0.0559
${\hat{p}}_{3}$	11.6895 ± 3.5051	10.4485 ± 2.2179
${\hat{p}}_{4}$	0.0320 ± 0.0380	0.0722 ± 0.0896
${\hat{p}}_{5}$	0.7983 ± 0.1320	0.8018 ± 0.2702

In order to investigate the effects of tDCS on underlying physiological mechanisms in the systemic energy metabolism, we compare mean estimated parameter values for tDCS and sham stimulation. Identified parameter ratios between tDCS and sham condition are shown and statistically significant values are highlighted in Table 2. Each ratio reflects the effect of tDCS on the respective physiological process described by the model parameter. To test if mean estimated parameter values upon tDCS and sham stimulation significantly differ, we conduct an analysis of variance, a two sample F-test. Hereby, a P-value < 0.1 was considered significant.

**Table 2:** Ratios of mean estimated parameter values for tDCS compared with sham condition

	${\hat{p}}_{1}$	${\hat{p}}_{2}$	${\hat{p}}_{3}$	${\hat{p}}_{4}$	${\hat{p}}_{5}$
**tDCS/sham**	2.45^a^	0.74^b^	0.89	2.26^c^	1.00

^a^Significance, P = 0.04, ^b^Significance, P = 0.08, and ^c^Significance, P = 0.03. Statistical analyses were performed by analysis of variance to identify main effects in experimental condition comparisons.

Glucose flux ${\hat{p}}_{1}$ across the blood brain barrier as well as cerebral energy consumption ${\hat{p}}_{4}$ significantly increase upon tDCS compared with sham stimulation. In contrast, the energy flux ${\hat{p}}_{2}$ from peripheral tissue into the blood significantly decreases in the stimulation condition. Insulin production rate ${\hat{p}}_{3}$ slightly decreases, and peripheral energy consumption ${\hat{p}}_{5}$ does not vary significantly between different experimental conditions.

The physiological interpretation of the obtained ratios reads as follows: TDCS excites cortical neurons. Our results verify that this leads to significantly increased cerebral energy consumption ${\hat{p}}_{4}$. Accordingly, our results reflect an increase in cerebral glucose uptake ${\hat{p}}_{1}$ from the blood into the brain crossing the blood brain barrier in response to excitation-induced energy consumption. This mechanism assures an adequate energy supply of the brain.

Moreover, we analyze cerebral energy supply on demand. Therefore, we calculate the ratio of energy flux ${\hat{p}}_{1}$ across the blood brain barrier to cerebral energy consumption ${\hat{p}}_{4}$. This yields ${\hat{p}}_{1}/{\hat{p}}_{4}$ ≈ 14.37 for tDCS compared to ${\hat{p}}_{1}/{\hat{p}}_{4}$ ≈ 1.95 for sham stimulation and the ratios significantly differ (P-value < 0.01). Our result reflects that the allocation mechanism of the brain is distinctly enhanced by transcranial stimulation. This reveals beneficial effects of neuronal activation on cerebral energy homeostasis and glucose metabolism.

Insulin production rate ${\hat{p}}_{3}$ is reduced upon tDCS. This reduction may be seen as enhanced allocation mechanism as well. With low blood insulin concentrations less energy is transported into the peripheral stores via the insulin-dependent glucose transporter GLUT4. Hence, available glucose can be transported into the brain via the insulin-independent transporter GLUT1.

Our results are in line with the hypothesis that the brain supplies itself with glucose in dependence of its own needs [3,9]. The allocation mechanism of the brain is activated with low high-energy phosphate content in the brain and the available energy is allocated to the brain.

Our findings explain the experimentally observed biphasic effect of tDCS on cerebral energy content, compare [25]. Neuronal excitation increases cerebral energy consumption ${\hat{p}}_{4}$ leading to a drop in high-energy phosphate content in the brain. Our results reveal a significant increase in glucose transport rate ${\hat{p}}_{1}$ across the blood brain barrier upon tDCS. Furthermore, cerebral energy supply on demand ${\hat{p}}_{1}/{\hat{p}}_{4}$ is enhanced. These observations explain the subsequent rise of cerebral high-energy phosphates above basal levels. The increase in cerebral glucose uptake in response to excitation-induced energy consumption thereby provides a conceivable explanation for the experimental observations. The allocation mechanism of the brain ensures cerebral energy supply.

Additionally, we find a significant decrease in energy flux ${\hat{p}}_{2}$ from peripheral tissue into the blood. This result is in line with the experimental measurements revealing suppression of the HPA system. Stress hormones such as cortisol induce gluconeogenesis mainly in the liver and thereby enhance the glucose flux from the resources into the blood. Reduced peripheral glucose release in conjunction with increased cerebral glucose uptake provides explanations for improved glucose tolerance.

Peripheral energy consumption ${\hat{p}}_{5}$ remains almost identical since the subjects are not physically active during examinations and therefore, the result meets the expectations. The obtained results are physiologically reasonable. Our investigations provide evidence for the experimentally observed changes in cerebral and peripheral energy metabolism upon tDCS. The results shed light on underlying physiological processes that are not measurable within the scope of a human experimental approach.

## Conclusions

For the first time, the relationship between neuronal brain activity and systemic energy metabolism was investigated in the experimental study [25] discussed and presented in Experimental study section. In the present study, collected experimental data are combined with the mathematical model [12] of the human whole body energy metabolism described in Brain-centered energy metabolism model section. Thereby, we predicted physiological relations and gained information about underlying physiological mechanisms. Additional validation of the model estimates on independent data is desirable and subject to further investigations.

Modeling approaches, especially in physiological applications, feature limitations in their compactness and in validity of the model functions. Energy homeostasis comprises a tremendous number of metabolites and complex physiological interrelations that are sufficiently relevant to be considered in the model equations. Moreover, most of these mechanisms are not yet sufficiently quantified to be used in mathematical models. To account for these limitations, we restricted our mathematical model to only include widely accepted and fundamental physiological relations. We showed that the whole-body energy metabolism can be realistically modeled and experimental data are reasonably predicted. Therefore, limited but relevant conclusions can be drawn from our findings. Nevertheless, in future work we aim to include further model refinements.

We are able to identify parameter values, for which our mathematical model reproduces experimentally acquired data, see Section 3.1. Our results verify the presented physiological mechanisms and validate the mathematical model. We for the first time developed a mathematical model predicting experimental data of cerebral and peripheral metabolites at the same time.

As mentioned above, the parameters of our mathematical model have a physiological interpretation. Analyzing the identified model parameters thus allow to draw conclusions about physiological mechanisms underlying the experimental data, see Effects of tDCS on underlying physiological mechanisms section. Thereby, we are able to explain effects in the experimental observations.

We experimentally observe a decrease followed by an increase in cerebral high-energy phosphates upon tDCS due to neuronal activation. However, underlying physiological mechanisms explaining this experimental findings remain unknown at this point. By the validity of our parameter estimation method we can draw the following conclusions: Firstly, cerebral energy consumption significantly increases upon tDCS compared with sham stimulation. This explains the initial drop in brain energy level. Secondly, our findings reflect a significant increase in glucose transport rate across the blood brain barrier. Thirdly, we observe an improvement of the allocation mechanism upon stimulation, which is expressed by an increase in the ratio ${\hat{p}}_{1}/{\hat{p}}_{4}$. Enhanced glucose uptake from the blood into the brain causes rising cerebral high-energy phosphates. Our findings support the hypothesis that the brain supplies itself with sufficient energy according to its needs [3]. Thereby, they support central aspects of the Selfish Brain Theory [9]. Fourthly, our findings elucidate effects of tDCS on peripheral energy metabolism, namely on glucose tolerance. Therefore, the present study gives information about effects of neuronal brain activity on systemic energy homeostasis in healthy humans.

In forthcoming investigations, we plan to investigate pathological conditions caused by deregulations in the energy metabolism such as obesity and diabetes mellitus. We want to identify model parameters of pathologic states thereby shedding light on defects causing metabolic diseases.

## Competing interests

The authors declare that they have no competing interests.

## Authors’ contributions

BG conceived and conducted the mathematical analyses, interpreted the results, wrote and edited the manuscript, and approved the final version for submission; KMO conducted the experimental study, interpreted the results, edited the manuscript, and approved the final version for submission; MC conceived and conducted the mathematical analyses, interpreted the results, edited the manuscript, and approved the final version for submission. All authors read and approved the final manuscript.
